# HIV Prevalence and Characteristics Among Patients With AIDS-Defining and Non–AIDS-Defining Cancers in a Tertiary Hospital in Kenya

**DOI:** 10.1200/GO.22.00360

**Published:** 2023-04-05

**Authors:** Diana Muturi, Sitna N. Mwanzi, Felix M. Riunga, Jasmit Shah, Reena Shah

**Affiliations:** ^1^ Department of Internal Medicine, Aga Khan University, Nairobi, Kenya; ^2^ Department of Haemato-Oncology, Aga Khan University, Nairobi, Kenya

## Abstract

Antiretroviral therapy (ART) has resulted in a higher life expectancy of persons living with HIV. This has led to an aging population at risk for both non–AIDS-defining cancers (NADCs) and AIDS-defining cancers (ADCs). HIV testing among patients with cancer in Kenya is not routinely performed, making its prevalence undefined. The aim of our study was to determine the prevalence of HIV and the spectrum of malignancies among HIV-positive and HIV-negative patients with cancer attending a tertiary hospital in Nairobi, Kenya.

**MATERIALS AND METHODS:**

We conducted a cross-sectional study between February 2021 and September 2021. Patients with a histologic cancer diagnosis were enrolled. Demographic data and HIV- and cancer-related clinical variables were obtained. HIV pretest counseling and consent were done, and testing was performed using a fourth-generation assay. Positive results were confirmed using a third-generation assay.

**RESULTS:**

We enrolled 301 patients with cancer; 67.8% (204 of 301) were female; the mean age was 50.7 ± 12.5 years. From our cohort, 10.6% (95% CI, 7.4 to 14.7, n = 32 of 301) of patients were HIV-positive with the prevalence of a new HIV diagnosis of 0.7% (n = 2 of 301). Of the HIV-positive patients, 59.4% (19 of 32) had a NADC. The commonest NADC was breast cancer (18.8%; 6 of 32), whereas non-Hodgkin lymphoma (18.8%; 6 of 32) and cervical cancer (18.8%; 6 of 32) were the most prevalent ADCs among HIV-positive patients.

**CONCLUSION:**

The prevalence of HIV infection among patients with cancer was twice the Kenya national HIV prevalence. NADCs comprised a larger percentage of the cancer burden. Universal opt-out HIV testing of patients attending for cancer care regardless of cancer type may facilitate early recognition of HIV-infected patients and aid in appropriate selection of ART and cancer therapies and preventive strategies.

## INTRODUCTION

The relationship between HIV and malignancies became evident early in the HIV epidemic, with Kaposi sarcoma (KS) being the first described AIDS-defining conditions.^1^ There are three cancers classified as AIDS-defining cancers (ADCs), and they include KS, cervical cancer, and non-Hodgkin lymphoma (NHL).^2^ These cancers are due to decreased immune surveillance caused by T-cell reduction by the HIV and subsequent immunosuppression. Nearly, a third of persons living with HIV (PLHIV) received a cancer diagnosis early on in the HIV epidemic before expanded access to antiretroviral therapy (ART).^3^ Non–AIDS-defining cancers (NADCs) comprise malignancies that occur among PLHIV and are not primarily due to the host's immune deficiency. These include Hodgkin lymphoma (HL), conjunctival squamous cell cancer, hepatocellular carcinoma, and anal, lung, and head and neck cancers. PLHIV are also at risk of sporadic cancers present in the general population, including those of the breast, colon, and prostate cancers.^4,5^

**CONTEXT**

**Key Objective**
What is the HIV prevalence among patients with cancer in Kenya and the spectrum of malignancies in this population? The HIV prevalence among patients with cancer in Kenya is undefined as diagnostic HIV testing of patients enrolling for cancer care is not offered routinely and cancers in HIV-positive patients are not well characterized outside AIDS-defining cancers.
**Knowledge Generated**
The prevalence of HIV among patients with cancer was 10.6%, which was two times higher than the national HIV estimate in the general population. Sixty percent of HIV-positive patients had a non–AIDS-defining cancer.
**Relevance**
Opt-out HIV testing of patients presenting to cancer units regardless of cancer type may facilitate early identification of HIV-infected patients who remain at risk of severe immunosuppression once treated with chemotherapy in the absence of antiretroviral therapy and appropriate opportunistic infection prophylaxis.

Cancer epidemiology among PLHIV has been influenced by the introduction of ART. A study in the United States on the proportion of cancers in PLHIV showed a greater than threefold reduction in ADCs with a concurrent triple increase in NADCs in the post-ART period.^6^ Longevity for PLHIV has increased with ART use, leading to a large cohort of older adults with a lifespan similar to the general population^7^ and who are at risk of NADCs in addition to ADCs.

Approximately 69% of PLHIV reside in Sub-Saharan Africa (SSA)^8^ where the use of ART started much later than the United States. Several factors make it difficult to assess cancer epidemiology and trends in this population and whether there is a similar shift from ADC to NADC.^9^ One such factor is that most African countries lack real-time population-based cancer registries, and those present have a bias toward the urban population.^10^ A cross-sectional medical review study at the Uganda Cancer Institute demonstrated a HIV prevalence of 23% among patients with cancer, which was three times higher than the HIV prevalence in Uganda.^11^ In this study, 42% of patients who had a documented HIV-positive status had a NADC. Another retrospective, descriptive study on HIV prevalence among patients with cancer in a surgical oncology unit in Guinea in the post-ART era showed a HIV prevalence of 2.1%.^12^ This prevalence was higher than the national HIV prevalence of 1.5%. This study also demonstrated a 79% proportion of NADCs. In Tanzania, a retrospective study in a zonal referral hospital among HIV-positive patients enrolled between 2009 and 2019 identified 9% of PLHIV who also had histologically confirmed cancers, 72% of which were NADCs.^13^

Knowledge of the HIV status of patients presenting for cancer care is important given this background. HIV-positive patients who are either unaware of their status or not on ART remain at risk of severe immunosuppression once treated with various cancer treatment modalities like chemotherapy.^14-16^ Starting ART and preventing further immunosuppression in addition to appropriate opportunistic infection prophylaxis have demonstrated good outcomes and increased survival among HIV-positive patients with cancer.^17^

In Kenya, the prevalence of HIV among patients with cancer is undefined and diagnostic HIV testing is not routine in our cancer care sites. Moreover, the spectrum of malignancies in PLHIV has not been well characterized outside of ADCs. Most studies have determined prevalence of individual cancers. The only study found describing various HIV–associated cancers was a laboratory-based study that reviewed 173 histologic blocks and reports. They demonstrated KS and conjunctival squamous cell cancer as the leading ADC and NADC in Kenya, respectively.^18^

The aim of this study was to explore the HIV prevalence among patients with cancer in a tertiary cancer center in Kenya, characterize the spectrum of malignancies in this population, and seek to answer the question of whether there was a similar observation of a shift from ADCs to NADCs. Knowledge of the proportion and range of HIV-associated cancers informs the extent of HIV cancer comorbidity on the health system and therefore facilitate planning of preventive strategies among PLHIV including smoking cessation, annual breast and cervical cancer screening, hepatitis B virus (HBV) and human papilloma virus (HPV) testing and vaccination, and age-appropriate cancer screening such as colonoscopy for those age older than 50 years. Early identification of HIV on presentation to cancer care sites and treatment with ART are contributing factors to treatment outcomes for patients with cancer who are also HIV-positive.

## MATERIALS AND METHODS

This was a cross-sectional study conducted at the Aga Khan University Hospital, Nairobi, between February 2021 and September 2021. Patients age 18 years and older with a histologically confirmed cancer diagnosis, who were with either known HIV-positive or with unknown HIV status, were eligible to participate in the study. Exclusion criteria included patients who declined HIV testing and those who did not give consent for study enrollment. Ethical approval was sought from the Aga Khan University, Nairobi Institutional Ethics Review Committee (Approval Number 2020/IERC-106). Approval was also obtained from National Commission for Science, Technology and Innovation. Written informed consent was obtained before entry into the study. Diagnostic HIV testing was performed using a fourth-generation HIV assay (Cobase 411/601 HIV Combi gen.2 assay) for those with unknown HIV status. Positive HIV results were confirmed with unigold rapid third-generation assay. Patients who tested HIV-positive received post-test counseling, and their attending clinician was informed. They were then linked to an infectious disease specialist for further management. Patients who were already known to be HIV-positive were also enrolled without further retesting. Data were collected on the basis of a data abstraction tool using the Research Electronic Data Capture (REDCap) platform (Vanderbilt and National Institute of Health)^19^ from electronic clinician and laboratory records. The tool captured patient demographic details, cancer characteristics, and HIV disease characteristics. Demographic details included age, sex, race, patient comorbidities, and smoking and alcohol history. HIV disease characteristics included the date of HIV diagnosis, date of ART initiation, current ART regime and history of defaulting ART, baseline CD4 count at HIV diagnosis, current HIV viral load, and known HBV status. The study used designated tumor (T), nodes (N), and metastases (M), TNM, one through four staging as per National Comprehensive Cancer Guidelines to stage each type of tumor. KS was staged as per AIDS Clinical Trials Group staging with designation of T for the extent of tumor, I for immune status, and S for severity of systemic illness. Binet staging was used for chronic lymphocytic leukemia (CLL). Early cancer was defined as TNM stages I and II, whereas late-stage cancer was defined as TNM stages III and IV. KS was designated as T0S0 or T1S0 when early, with any other designation classified as late stage.^20^ Binet C for CLL was set as late stage. Functional status was evaluated with the Eastern Cooperative Oncology Group (ECOG) score 0 through 5. Poor functional status was assigned for an ECOG score >2.^21^ HIV viral load obtained while on ART was grouped into three categories: <50 copies/mL, 51-200 copies/mL, and >200 copies/mL. Patients with a viral load of >200 copies/mL were classified as having treatment failure. The REDCap data were checked for completion and assessed for any errors. All the data were deidentified for analysis. Continuous variables were summarized using median and IQR, and categorical variables using frequency and percentages. Cross-tabulations with χ^2^ or Fisher's exact test were performed to relate variables of interest. All the analyses were performed using SPSS version 23, and a *P* < .05 was considered statistically significant wherever applicable.

## RESULTS

### Sample Characteristics

Three hundred one patients with cancer were enrolled in the study. The median age at cancer diagnosis was 51.0 (IQR, 42.0-59.0) years, 67.8% of the study population were female, and majority (92.0%; n = 277) were of African origin. The most prevalent comorbid condition among the participants was hypertension 26.6% (n = 80) followed by diabetes at 13.3% (n = 40). A majority of respondents (86.4%, n = 260) had never smoked in their lifetime, whereas 12.6% (n = 38) were former smokers and only 1.0% (n = 3) were current smokers. 64.8% (n = 195) of the respondents had never consumed alcohol, whereas 25.6% (n = 77) and 9.6% (n = 29) were former and current users, respectively (Table 1).

**TABLE 1:**
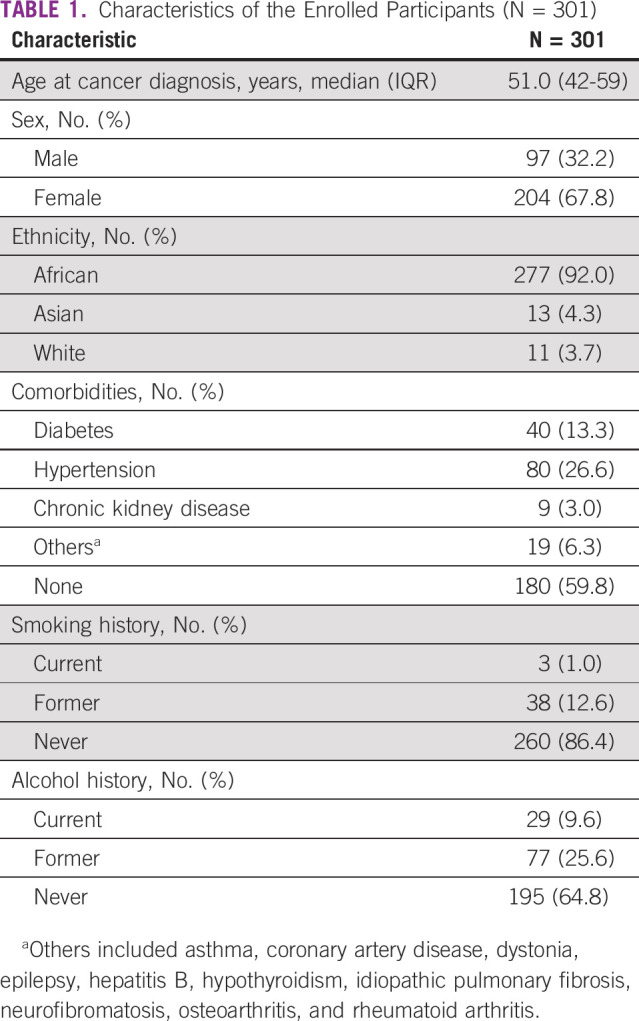
Characteristics of the Enrolled Participants (N = 301)

### HIV Characteristics

The results indicate that the prevalence of HIV among those with known HIV-positive and unknown HIV status was 10.6%, representing 32 study participants. Thirty participants were known to be HIV-positive, whereas two were newly diagnosed. The median age of HIV-positive patients was 49 (IQR, 44-57) years, 65.6% of whom were female (n = 21 of 32).

Among HIV-positive patients, 93.9% (n = 30 of 32) had a HIV-1 viral load test done. Of these, 76.7% (n = 23 of 30) had a viral load of <50 copies/mL and 6.6% (n = 2 of 30) had a viral load in the range of 51-200 copies/mL, whereas the remaining 16.7% (n = 5 of 30) had a viral load above 200 copies/mL. Only 50.0% (n = 16 of 32) had CD4 counts recorded at HIV diagnosis. For these patients, the median CD4 count was 73.0 cells/mm^3^ (IQR, 40.0-157.0). The most common ART regimen was tenofovir/lamivudine/dolutegravir (TDF/3TC/DTG), whereas the least common were tenofovir/lamivudine/efavirenz (TDF/3TC/EFV), tenofovir/lamivudine/atazanavir/ritonavir (TDF/3TC/ATV/r), and tenofovir/emtricitabine/efavirenz (TDF/FTC/EFV), each used by 3.2% of the patients. One patient had a new HIV diagnosis and was yet to start ART. Only 12.5% (n = 4) of patients had a history of defaulting on ART. Five percent (n = 1 of 19) had a coinfection of HIV and hepatitis B (Table 2).

**TABLE 2:**
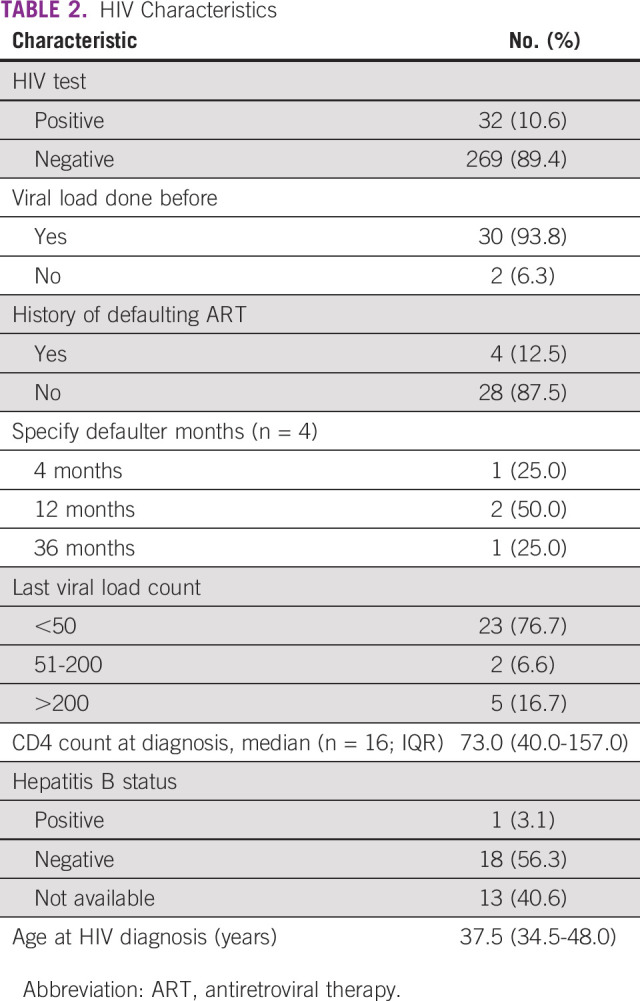
HIV Characteristics

### Cancer Characteristics

Table 3 summarizes cancer characteristics and cancer sites of the study population. Breast cancer was the most frequent cancer diagnosis, 33.6% (n = 101). Forty-one percent (n = 126) had stage IV cancer, 30.2%, 13.6%, and 2.7% had stages III, II, and I, respectively. Five of the participants had cancers with different staging systems including BINET C (n = 4) in CLL and T1I1S1 in the only patient with KS. The remaining 30 patients had cancers, with no standardized staging system including multiple myeloma and acute leukemia. The prevalence of late-stage (stage III and IV) cancer was 81.6% (95% CI, 76.4 to 86.1). Sixty-six percent (n = 200) had an ECOG score of 1 and 21.3% had a score of 0, whereas 9.6%, 2.0%, and 0.7% had scores of 2, 3, and 4, respectively.

**TABLE 3:**
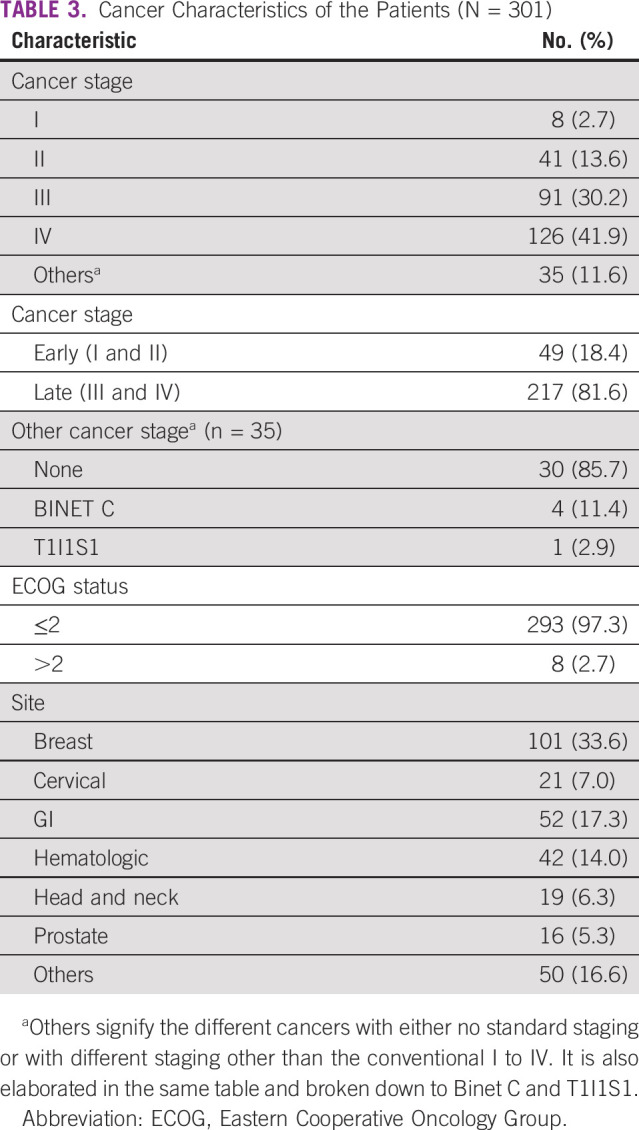
Cancer Characteristics of the Patients (N = 301)

### Types of Cancers Among HIV-Positive Patients

**FIG 1:**
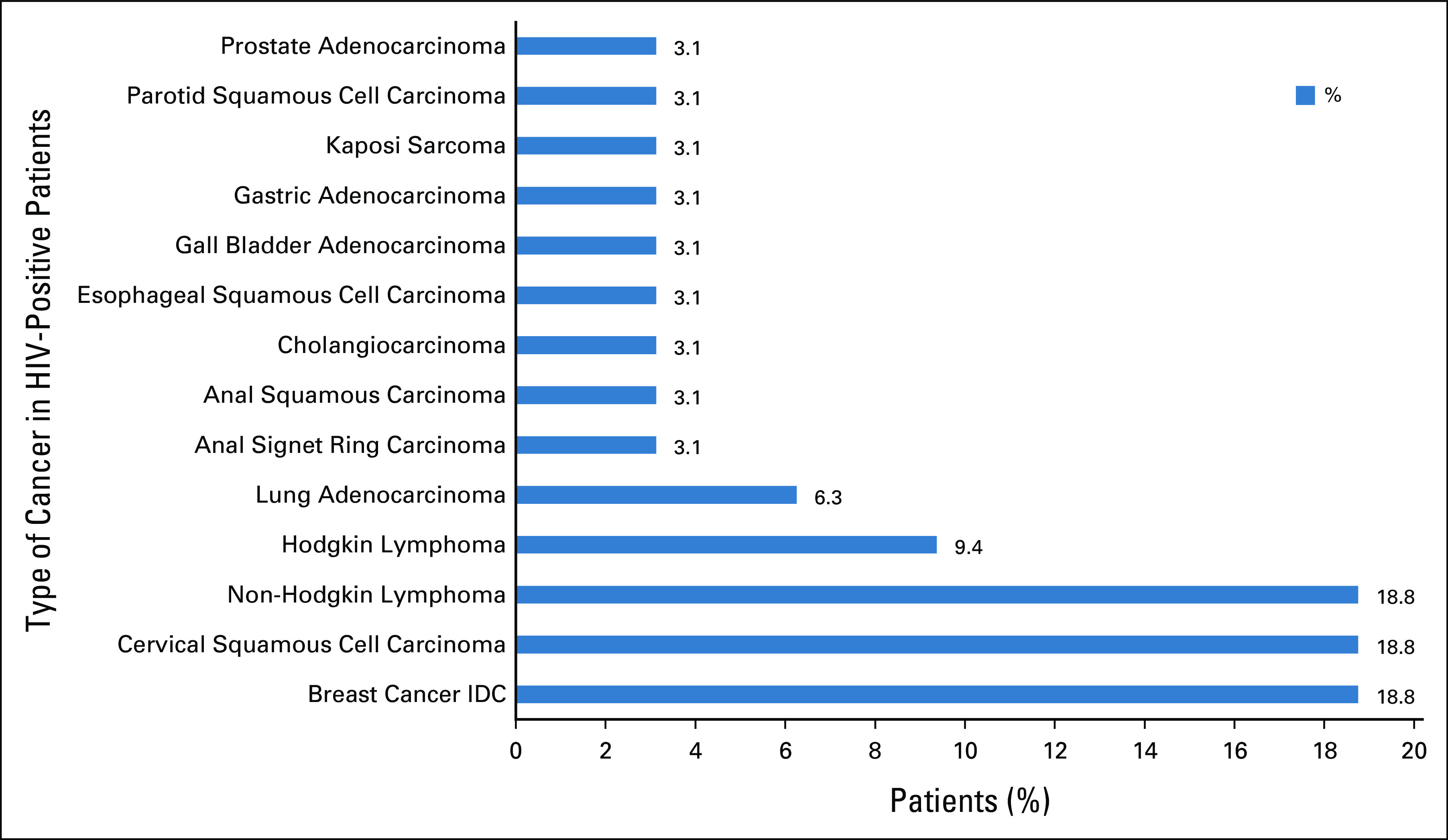
Spectrum of malignancies among HIV-positive patients (n = 32). IDC, invasive ductal carcinoma.

Different types of cancers diagnosed among the 32 HIV-positive patients are shown in Figure 1. The most common NADC among HIV-positive patients was breast cancer invasive ductal carcinoma (18.8% n = 6). 9.4% were diagnosed with HL, whereas 6.3% had lung adenocarcinoma. The GI cancers included anal signet ring carcinoma, anal squamous carcinoma, cholangiocarcinoma, esophageal squamous cell carcinoma, gall bladder adenocarcinoma, and gastric adenocarcinoma, each diagnosed in one patient. The most common ADCs were cervical cancer and NHL in equal proportions (18.8% [n = 6]). Among the patients with NHL, only one patient had Burkitt's lymphoma subtype. Of interest was the low frequency of KS (3.1% [n = 1]) in the study.

**FIG 2:**
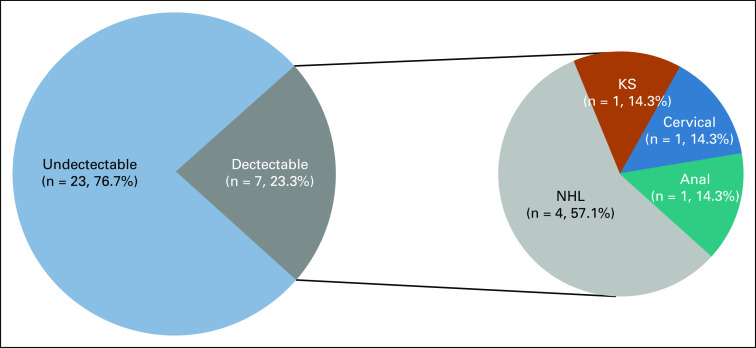
HIV viral load by cancer type. KS, Kaposi sarcoma; NHL, non-Hodgkin lymphoma.

Twenty-three percent (n = 7) of HIV-positive patients had a detectable viral load above 50 copies/mL, six of whom had ADCs that included four with NHL and one each with KS and cervical cancer. There was only one patient with NADC (anal cancer) who had a detectable HIV viral load (Fig 2).

**FIG 3:**
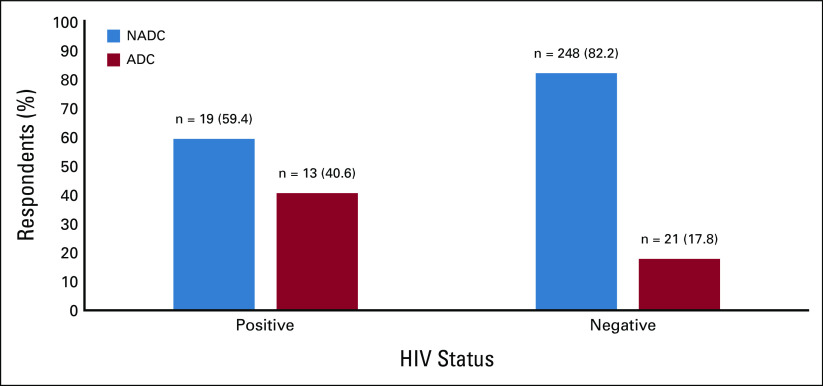
Proportion of NADCs and ADCs by HIV status. ADC, AIDS-defining cancer; NADC, non–AIDS-defining cancer.

Of the 32 HIV-positive participants, 59.4% (n = 19) had a NADC, whereas 40.65% (n = 13) had an ADC. The prevalence of NADC and ADC among HIV-negative patients was 248 (82.2%) and 21 (17.8%), respectively (Fig 3).

## DISCUSSION

Our study documented a HIV prevalence of 10.6%, which was twice the national HIV prevalence among an adult general population age 15-64 years in the 2018 Kenya population–based HIV impact assessment (KENPHIA) survey.^22^ When stratified by age group, the HIV prevalence of the general population in the KENPHIA survey aged 50-54 years was 9.2% comparable with the 10.6% prevalence of the enrolled participants with a median age of 51 years.

This was similar to the nine percent prevalence of HIV-cancer comorbidity in a study by Mremi et al^13^ in a Tanzanian zonal hospital but lower than what was described in a Ugandan study, which demonstrated a 23% prevalence.^11^ The lower prevalence in our study may be influenced by the patient catchment of a private institution with higher socioeconomic status and may not reflect that in a middle or lower socioeconomic population.

Identification of HIV-cancer comorbidity through diagnostic HIV testing of patients attending hemato-oncology units is not offered routinely. The higher HIV prevalence demonstrated in this population compared with the national HIV prevalence implies the need to perform diagnostic HIV testing in patients with cancer presenting for enrollment. HIV-positive patients who are either unaware of their status or not on ART remain at risk of severe immunosuppression once treated with chemotherapy.

Sixty percent of the cancers among HIV-positive patients in the study were NADCs, demonstrating a shift in cancer epidemiology from ADCs to NADCs in PLHIV similar to that observed in high-income countries with ART availability.^6,23^ A study performed a decade ago at Kenya's largest referral hospital showed that ADCs dominated the cancer burden among PLHIV in Kenya.^18^ Since then, Kenyan HIV treatment guidelines have changed to recommend universal testing and treatment for all who were found to have HIV with ART as recommended by the WHO.^24^ This is projected to result in higher survival rates among PLHIV and a large cohort of older HIV-positive adults with cancer risks similar to the general population and not necessarily cancers associated with profound immunosuppression attributable to HIV infection.

The changing trend and shift toward NADCs among PLHIV in SSA were demonstrated in Guinea and Tanzania with the 79% and 72% prevalence of NADCs, respectively, underscoring the need to perform diagnostic HIV testing in patients with cancer irrespective of the cancer type.^12,13^ Among the NADCs, the commonest malignancy among HIV-positive patients in the study participants was breast cancer. The predominance of breast cancer among NADCs has been shown in SSA. A study performed in Soweto, South Africa, among patients with breast cancer demonstrated a HIV prevalence of 19.7%.^25^ The high breast cancer prevalence in our study could be because it is also the most frequent cancer among Kenyan women. As per the 2020 GLOBOCAN estimates, breast cancer had the highest incidence of 16.1% (n = 6,799 of 42,116) in Kenya.^26^ Contrary to the present study, Mpunga et al^27^ showed that conjunctival, HL, and anal malignancies were the most frequent NADCs in Rwanda. HL, lung, and anal malignancies and hepatocellular carcinoma were the most common NADCs in high-income countries.^6^

Cervical cancer and NHL were the most prevalent ADCs in our study, occurring in equal proportions. These findings were comparable with those described in other SSA countries.^9,13,27-30^ ADCs, which accounted for 40% of all cancers among HIV-positive patients, are a result of advanced immunosuppression suggested by the median CD4 count well below 200 cells/ul at HIV diagnosis among the study population. Advocacy is needed for routine HIV testing to ensure early ART initiation to reduce the ADC burden among PLHIV. There was a low rate of KS in our study with only one case identified. This is in contrast to various SSA studies, which showed KS as the most common ADC among PLHIV.^13,18,27,31^ In the present study, a majority of patients who were HIV-positive were virally suppressed and the frequency of ADCs such as KS would be expected to be lower although there was no record of current CD4 counts. The ART coverage was nearly 100% among PLHIV in our study in line with the 90% WHO recommendation.^24^ Among the HIV-positive patients, 18% had not achieved HIV viral suppression, placing them at potential risk for further immune suppression while receiving chemotherapy. Cross-referral among oncologists and infectious disease specialists would assist in optimal control of both illnesses, thereby improving survival and outcomes for these patients.

Our study limitations included inability to determine any cause-effect relationship or HIV associations with individual cancer types in view of the cross-sectional nature of the study. The design also did not allow comparison of the post-ART with pre-ART period. Our study gave a HIV prevalence and spectrum of malignancies at a private tertiary teaching hospital that may not be generalizable to the rest of the country as it might have been biased by patient catchment with different health seeking behavior patterns and might not have reflected the prevalence in a lower socioeconomic population. Potential for selection bias might have misstated the estimated prevalence of HIV among patients with cancer.

In conclusion, to our knowledge, this is the first study on HIV prevalence among patients with malignancies in Kenya and the study demonstrated prevalence that was two times higher than the national estimate. NADCs comprised a larger percentage of the cancer burden among PLHIV, suggesting that universal opt-out HIV testing of patients presenting to hemato-oncology units regardless of cancer type may facilitate early identification of HIV-infected patients and aid in appropriate selection of ART and cancer therapies. The burden of ADCs among PLHIV is still significant despite widespread availability of ART, suggesting that efforts need to be continually placed for early identification of HIV infection and early ART commencement before severe immunosuppression sets in. The most prevalent cancers among HIV-positive patients were breast cancer, cervical cancer, and NHL. Most of these cancers offer an opportunity for deployment of preventive strategies such as HPV vaccination to prevent cervical cancer and mammography for detection of early breast cancer among PLHIV. We recommend a Kenyan population HIV/AIDS-cancer match study to obtain an accurate HIV prevalence among patients with cancer for optimal management for patients with the two comorbidities. Universal opt-out HIV testing should be offered to patients on enrollment to cancer care units to offer an optimal ART treatment regimen and opportunistic infection prophylaxis and plan for chemotherapy so as to avoid further immunosuppression that may occur on initiation of chemotherapy. Through the study, a cohort of HIV-positive and HIV-negative patients with cancer was established and can act as a foundation for further studies that can be performed in the subject area of HIV-associated cancers including long-term follow-up and whether overall survival and outcomes are comparable between the two groups.
